# Editorial Notes

**Published:** 1906-12

**Authors:** 


### Editorial Notes.

**University College, Bristol. Faculty of Medicine.**

THE annual distribution of prizes to the students took place on October 2nd, 1906, under the chairmanship of the Right Hon. Lewis Fry, who congratulated the Dean of the Medical Faculty on his having obtained for the occasion the services of so distinguished a person as Professor Alexander MacAlister, of the Cambridge University, whose services in many departments of science were so universally recognised. Professor MacAlister's address is of especial interest in connection with the proposal to establish a new University of Bristol, and it commenced with the following remarks:—

"I may lay claim to have a certain shadowy connection with Bristol, as by accident I was born in the city of Dublin, a city which in 1172 King Henry II. presented to Bristol *ad inhabitandum*. I cannot, however, claim kindred either with the first set of Bristolian colonists, who were unfortunately wiped out by their truculent aboriginal neighbours on Easter Monday, 1209, or with their successors, who in the reign of King John repeopled the city on the banks of the Liffey.

"In one respect the daughter city, after the manner of daughters, has progressed faster than her mother. In 1892 she celebrated the tercentenary of the foundation of her University, whereas in this city I understand that your University is now in the throes of birth. Not that you have been without a centre of education all this time, for I believe that your Medical School has had a somewhat discontinuous existence since Mr. Page began his public lectures on Anatomy in 1746. It is encouraging to hear that at present the movement which is leading to the development of local Universities has reached this western city, and is likely to bear fruit. You have here all the conditions which fit your city to be the seat of a successful and active University—a sufficient population to draw upon, a large commercial and manufacturing centre, an ancient and famous seaport, and an abundance of public spirit among your leading citizens.

"As long ago as 1830 your townsman, Mr. Prichard, in a letter published in *Felix Farley's Journal*, advocated the foundation of a University here, and pointed out how much England was behind Scotland in the matter of local Universities, expressing his belief that the prosperity of the latter country and the success of her sons in all lands were for the most part due to its proportionally numerous and accessible educational institutions. The five western counties and shires from which you may expect to draw your students include nearly two millions and a half of people, while the nine northern counties which constitute the basin of drainage for Aberdeen have a population somewhat under one million; and yet the northern city of Aberdeen has been during the last week celebrating the four hundredth birthday of its flourishing University, whilst St. Andrews, the centre of a much smaller district, has also a University which in five years will be half a millenium in age.

"Bristol University will doubtless be in some respects more happily situated than the University in your daughter city, which has been continually harassed through its three hundred years of turbulent history with contentions due to race prejudices and religious animosities — troubles which, instead of abating, have in late years gathered so ominously that they are at present about to be treated by that sovereign constitutional panacea, a Royal Commission.

"The birth of so many new Universities may be one of the most hopeful signs of national vitality and intellectual progress if—and it is a large 'if'—they are to be centres of research as well as centres of instruction. There is in some quarters at present a spirit of cheap and purblind utilitarianism, owing to which there is some danger that some at least of the new Universities may have ideals which make them become little better than technical schools, and whose energies are wholly occupied with the mechanical task of turning out a certain number of graduates more or less equipped for the routine of professional, commercial, or industrial life, and, as in some places, the maintenance of practical departments depends chiefly on fees, there is a temptation towards a competition downwards in order that larger classes may be secured, success being reckoned by the counting of heads. If it is to fulfil the function for which it should exist, the University should be much more than a mere technical and professional school. The technical school is an important place of education—would we had more of them!—but the University training should be broader and of a different grade, and the institution should be equipped and maintained so that its resources shall be employed not only in the teaching of old truth, but in the discovery of new.

"This is not merely the doctrinaire view of an amateur educationalist. It is of enormous practical, national importance that provision should be made for the furtherance and encouragement of scientific research. The discovery which is only a curiosity to-day may be the groundwork of an important industry and a source of national wealth next year."

**Proposed University for Bristol.**

It is impossible to avoid drawing a contrast between Aberdeen and our own city in University matters. The northern city, with a population at that time of about 4,000, received in 1494 its first charter for University teaching by the foundation of King's College, whose quater-centenary has just been celebrated. A second University was established in 1593 for about 7,300 inhabitants, and for two centuries these two University Colleges with full University powers continued to maintain a more or less independent existence. Of late years they have worked together as one combined University, supplying the highest University advantages to a city whose present population amounts to about 160,000, whereas we in Bristol, with a population of more than twice that number, have as yet no University at all.

The proposed scheme was much discussed at a farewell dinner given on October 12th to Professor Morris W. Travers, F.R.S., shortly before his departure to India.

Dr. Michell Clarke remarked that towards the end of the nineteenth century the numerous public buildings devoted to educational purposes was very striking, and he hoped that the beginning of the twentieth century would be characterised by a still further development of this activity, and that they would crown their primary and secondary school system by a University. He trusted that future years would find a permanent memorial in a fine building emblematic of the work which their age and generation did at the time for the city. He wondered why so old a city as Bristol had not had a University before; it was not a wonder they were seeking one now.

Professor Travers pointed out that a University should have as its aim the highest advancement of learning. There was every need to form definite views on this question, and Bristol differs from many other large centres in which Universities had been recently founded. The question might well be asked, "In what should Bristol specialise?" Could they not have a great faculty of medicine? For that they seemed well fitted; and if they increased their students sevenfold, their medical charities would, he thought, provide sufficient facilities for that part of their training. The medical department was one of the most striking features of the University College. Then Bristol had industries wanting engineering, chemistry, applied sciences, electricity, technics, and he might ask about their commerce. They had the world's commerce. Could they not cater for that and supply something—which would be defined after experiment and inquiry—which would interest the greatest part of Bristol's commerce? They had great merits; they had Cabot and Colston. Chairs of economic geography were rare; there was a subject for Bristol to take up. Chairs for commercial economics were not common. What two names could be better attached to these chairs than Cabot and Colston? They must find out what they wanted and put it before the public before the public would help in the movement. Those connected with the college in the past had tried to carry out the ideal he had named—to carry learning to its highest limits. What they wanted was not only to have, but to keep men, for without them they could not make a University. When their scheme assumed more definite shape he had little doubt the City

Council would aid them, and he hoped too that the Merchant Venturers would take in it the leading part they always had in Bristol affairs.

**Aberdeen University Quater-Centenary.**

An old graduate of the grey city of the North, Dr. Rattray of Frome, sends us the following account of the celebrations connected with its quater-centenary, "which are not likely to be easily forgotton by the vast assemblage of delegates, graduates and guests at the granite city during the third week of last September. Such a concourse of the *élite* of Science and Art of the whole world is not often to be witnessed. All the Universities and educational centres of importance in the kingdom sent representatives, also the Colonies, America, Japan, and nearly every European State. Bristol was well represented by Sir Edward Fry, Professor Lloyd Morgan, Dr. Shingleton Smith, Dr. Fletcher, and others.

"The crowning function of the week's entertainment was the opening of the new buildings, constituting the front of Marischal College. This is the section of Aberdeen University devoted to the Faculties of Science and Medicine. The increase of class-rooms and laboratories, the completion of the Mitchell Hall and the great Mitchell Tower, have in later years rendered the college a most imposing building, but it had long been felt how unworthy was the approach—merely a low archway in a line of old shops and houses and slumlike passages. Now these buildings are all cleared away, and Marischal College can boast of a magnificent façade somewhat resembling in structure the front of the Houses of Parliament in Westminster Palace Yard.

"Thursday in the quater-centenary week was the great day, when Their Majesties the King and Queen honoured the city of Aberdeen with their presence, and graciously performed the opening ceremony. Who will ever forget that brilliant scene, when some five thousand individuals, most of them displaying brightly-coloured academic robes, sat patiently awaiting the advent of their gracious Sovereign and his Royal Consort? The hour timed for the arrival of royalty was one o'clock, and as the possessors of tickets had to be in their appropriated places not later than ten o'clock, there were three hours of expectancy to be endured. How short that brief period of three hours! Long ago within the building three hours was the time allotted to a professional examination, the paper on Advanced Anatomy, or on Practice of Medicine. What a contrast, those happy hours gaily passing with jokes and stories and memories grave and gay!

"Just before the appointed hour a fanfare of trumpets heralded the arrival of the royal party. For a brief moment a breathless pause, then a right, lusty, rousing cheer as the characteristic form of King Edward VII., resplendent in field-marshal uniform, accompanied by Her Majesty the Queen, appeared on the daïs in front of the vast throng.

"The interesting ceremony was not of long duration. The address read to the King by Principal Marshall Lang, the King's reply, and then in sonorous tones, with both arms extended and facing the assembled company, His Majesty concluded with the words, 'I now declare these new buildings open.' Prayer offered up by the Professor of Church History, and the Old Hundredth Psalm sung by all assembled, accompanied by the band of the Royal Scots Greys of Edinburgh Castle, and the great ceremony passed into history.

"It has been said that if you wish to reach an Englishman's heart it must be by way of his stomach, possibly by the pneumogastric nerve. This would seem to have held good at the quatercentenary function in the far North. The great Strathcona banquet held on the evening of the Thursday was from first to last a gigantic success. The arrangement of guests at table so that graduates of the same year should be grouped together was a very happy idea, and the lettering and numbering of the tables with the letter and number of each guest affixed to his name in the list presented with each menu conveniently enabled old friends to recognise each other. The popular thing before the banquet began was to get as many signatures of old friends as could be written on the flyleaf of the magnificently elaborated menu.

"Throughout the week the other items of the entertainment may just be mentioned:—The procession to the Strathcona Hall and the reception of delegates on the opening day; the conferring of honorary degrees; the reception at the City Art Gallery; the afternoon reception at King's College; the evening reception at Marischal College, to view the interior of the new buildings. Besides these the Aberdeen Medico-Chirurgical Society gave splendid hospitality—a couple of dinners and a most delightful excursion by rail to Dunnottar Castle. There were also class reunion dinners, students' torchlight procession, banquet by the Lord Provost, magistrates and Town Council, students' ball, and students' symposium.

"Perfect weather favoured a week of unsurpassable enjoyment. The quater-centenary was an event of transcendent pleasure and interest. We saw and learned the great advances since 'our time'—close on a quarter of a century—and the present, between the 'then' and 'now.' Clearly Aberdeen is well to the fore in the great struggle for supremacy in the teaching of the Healing Art. Brains and perseverance enhance the possession of those fully-equipped laboratories and spacious and elegant class-rooms. Well may one rejoice that one's Alma Mater has seen such a day of encouragement, and it is no small satisfaction that the celebrations were so much enjoyed by eminent critics from almost every quarter of the globe. Long may the University flourish, and long too may last those handsome grey towers and battlements which will ever perpetuate the illustrious names of Mitchell and Strathcona, and which also will ever bring back to the memory of those who are privileged to revisit their native town the glorious spectacle of that eventful day when a new era of activity sprang upon the celebrated University by the inauguration of the new buildings by His Majesty the King."

